# Cytoprotective effect of neuropeptides on cancer stem cells: vasoactive intestinal peptide-induced antiapoptotic signaling

**DOI:** 10.1038/cddis.2017.226

**Published:** 2017-06-01

**Authors:** Konduru S Sastry, Aouatef Ismail Chouchane, Ena Wang, George Kulik, Francesco M Marincola, Lotfi Chouchane

**Affiliations:** 1 Laboratory of Genetic Medicine and Immunology, Weill Cornell Medicine-Qatar, Education City-Qatar Foundation, Doha 24144, Qatar; 2 Division of Translational Medicine, Sidra Medical and Research Center, Doha 26999, Qatar; 3 Department of Cancer Biology, Wake Forest University School of Medicine, Winston Salem, NC, USA; 4 College of Science, Alfaisal University, Riyadh 11533, Saudi Arabia; 5 Research Division, Sidra Medical and Research Center, Doha 26999, Qatar

## Abstract

Cancer stem cells (CSCs) are increasingly considered to be responsible for tumor initiation, metastasis and drug resistance. The drug resistance mechanisms activated in CSCs have not been thoroughly investigated. Although neuropeptides such as vasoactive intestinal peptide (VIP) can promote tumor growth and activate antiapoptotic signaling in differentiated cancer cells, it is not known whether they can activate antiapoptotic mechanisms in CSCs. The objectives of this study are to unravel the cytoprotective effects of neuropeptides and identify antiapoptotic mechanisms activated by neuropeptides in response to anticancer drug treatment in CSCs. We enriched and purified CSCs (CD44^+/high^/CD24^−/low^ or CD133^+^ population) from breast and prostate cancer cell lines, and demonstrated their stemness phenotype. Of the several neuropeptides tested, only VIP could protect CSCs from drug-induced apoptosis. A functional correlation was found between drug-induced apoptosis and dephosphorylation of proapoptotic Bcl2 family protein BAD. Similarly, VIP-induced cytoprotection correlated with BAD phosphorylation at Ser112 in CSCs. Using pharmacological inhibitors and dominant-negative proteins, we showed that VIP-induced cytoprotection and BAD phosphorylation are mediated via both Ras/MAPK and PKA pathways in CSCs of prostate cancer LNCaP and C4-2 cells, but only PKA signaling was involved in CSCs of DUVIPR (DU145 prostate cancer cells ectopically expressing VIP receptor) and breast cancer MCF7 cells. As each of these pathways partially control BAD phosphorylation at Ser112, both have to be inhibited to block the cytoprotective effects of VIP. Furthermore, VIP is unable to protect CSCs that express phosphorylation-deficient mutant-BAD, suggesting that BAD phosphorylation is essential. Thus, antiapoptotic signaling by VIP could be one of the drug resistance mechanisms by which CSCs escape from anticancer therapies. Our findings suggest the potential usefulness of VIP receptor inhibition to eliminate CSCs, and that targeting BAD might be an attractive strategy for development of novel therapeutics.

Most tumors harbor a very small subset of specialized cells, named as cancer stem cells (CSCs) or tumor initiating cells, that are at least in part responsible for the initiation, progression and relapse of cancer. These CSCs display self-renewal ability to maintain the population of tumorigenic cells and plasticity to produce multiple cell types that comprise the tumor. The detection of CSCs in many tumors together with the emerging scientific support for the CSC hypothesis greatly revolutionized our outlook on the carcinogenesis and chemotherapy.

Another important property of CSCs is their ability to display resistance to anticancer drugs.^1, 2, 3, 4, 5^ Several conventional anticancer drugs can eliminate most of differentiated cancer (DC) cells, but they fail to target CSCs, resulting in tumor relapse.^6, 7^ This failure is associated with the activation of antiapoptotic mechanisms in DC cells and CSCs.^8^ Several growth factors, cytokines and neuropeptides activate survival pathways in tumor cells.^9, 10, 11, 12, 13^ One of the widely studied antiapoptotic mechanisms contributing to the drug resistance is the dysregulated expression or phosphorylation of pro- and antiapoptotic Bcl2 family proteins. We and others showed that CSCs express elevated levels of antiapoptotic proteins of Bcl2 family.^12, 14, 15^ BAD (Bcl2-antagonist of cell death) is a member of the BH3-only proapoptotic Bcl2 family protein that controls cell survival through its phosphorylation on at least two different sites, Ser112 and Ser136.^16, 17, 18^ We showed that while dephosphorylated BAD can promote apoptosis, phosphorylation of BAD by EGF or estradiol can protect CSCs from apoptosis.^12^

Neuropeptides, which can act as neurotransmitters and hormones, are small regulatory molecules that are widely distributed in the body and regulate diverse physiologic processes via G-protein coupled receptors. They can act as autocrine or paracrine growth factors in tumor cells. Several neuropeptides such as vasoactive intestinal peptide (VIP), bombesin (Bom), gastrin releasing peptide (GRP), calcitonin (Calci), parathyroid hormone-related peptide (PTHRP) and endothelin (Endo), as well as a neurotransmitter serotonin (Sero) have been shown to increase the proliferative capacity of cancer cells.^19, 20, 21^ In addition, some of these neuropeptides can increase the invasion and migration of cancer cells leading to metastasis.^22, 23, 24^ Because of the extensive role in carcinogenesis, VIP has drawn a special focus. Specifically, an elevated expression of VIP receptors (VIPR) has been found in several cancers.^25, 26, 27, 28, 29^ We showed that VIP protects cancer cells from apoptosis,^9^ and VIPR antagonists could inhibit the proliferation of cancer cells and reduce the growth of tumor xenografts.^30^

Although much is known about the potential roles of neuropeptides in DC cells, it is not known whether they can induce similar antiapoptotic mechanisms that contribute to drug resistance in CSCs. This prompted us to explore the potential role of VIP and other neuropeptides in CSCs. We started our investigation by assessing the antiapoptotic activity of VIP in CSCs and then extended these experiments using other neuropeptides. As we found that only VIP could protect CSCs from anticancer drug-induced apoptosis, we investigated the signaling pathways activated by VIP.

## Results

### Expression of VIPR1 in cancer cell lines and breast cancer tumors

**Figure 1:**
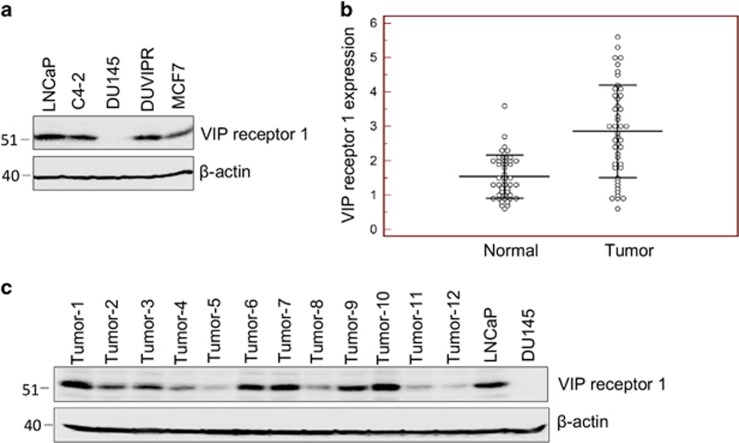
Expression of VIP receptor in cell lines and tumors. (**a**) Cell lysates of indicated cells were western blotted, and the blot was developed with anti-VIPR1 antibodies. Equal loading was assessed by *β*-actin antibodies. (**b**) VIP receptor expression was measured by quantitative real-time PCR on malignant and non-malignant tumor adjacent normal tissue from breast cancer patients. In about 60% tumors VIPR1 expression was more than non-malignant tissue. (**c**) Protein was extracted from breast cancer tumors and 50 *μ*g protein was western blotted, and the endogenous VIPR1 expression was detected

VIP binds to VIPR and performs a wide variety of functions in cancer and normal cells. We first determined the expression levels of VIPR1 in various cancer cells. LNCaP and C4-2 prostate cancer cell lines and MCF7 breast cancer cells expressed comparable levels of VIPR1 (Figure 1a). However, DU145 cells lack the VIPR1 expression and therefore acted as a negative control in our experiments. Furthermore, we introduced *VIPR1* gene into DU145 cells through lentiviral vector, and the transformed cells were selected in puromycin. The clone, DUVIPR, that expressed physiological levels of VIPR1 (compared with other prostate cancer cells) was used throughout our experiments.

To confirm whether VIPR1 is also expressed in patient tumors, we compared VIPR1 mRNA expression in breast tumors and in tumor adjacent normal breast tissue by quantitative real-time PCR. VIPR1 mRNA expression was detectable in more than 90% tumors (41 of 45). Moreover, in 27 of 45 samples (60%), the VIPR1 expression was higher in tumor tissue than in normal tissue (Figure 1b). Furthermore, we isolated total protein from breast tumors and western blotted using VIPR1 antibody. Our results (Figure 1c) confirm varying expression levels of VIPR1 in breast tumors. Together, these results suggest that VIPR1 expression is maintained in breast tumors and might have a potential role in carcinogenesis.

### Enrichment, FAC-sorting and characterization of CSCs

**Figure 2:**
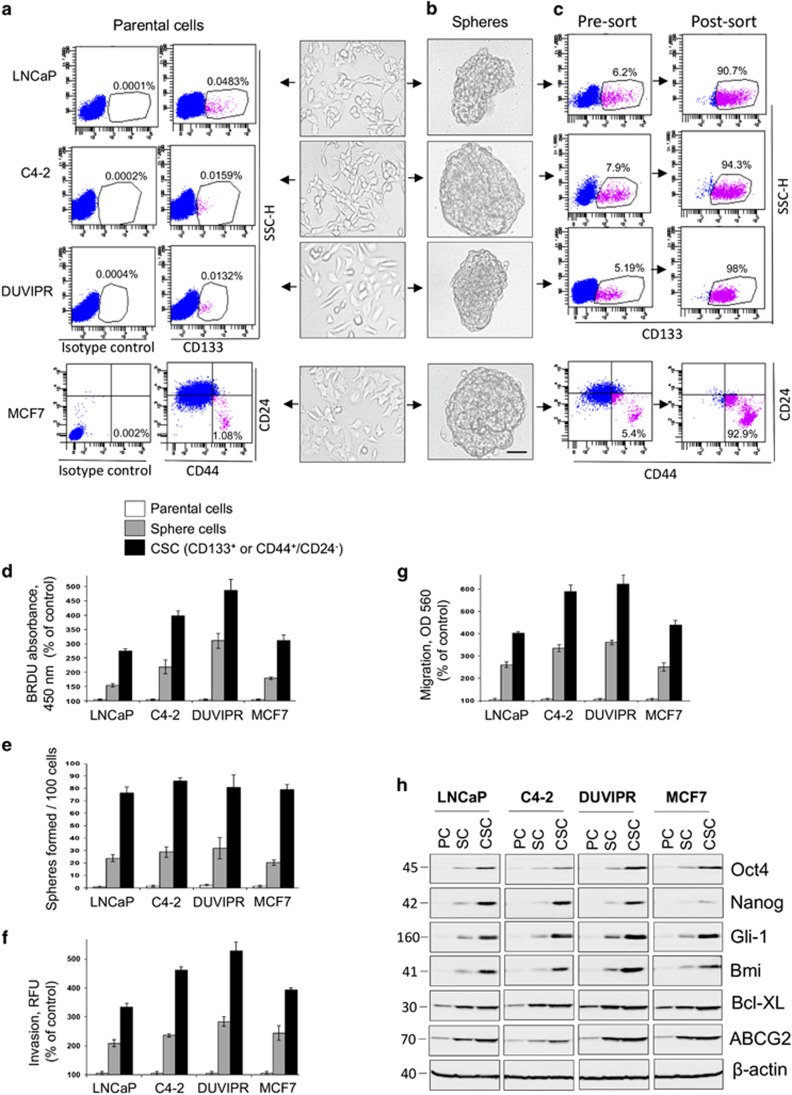
Enrichment, purification and characterization of CSCs. (**a**) Identification of CSCs: trypsinized parental prostate cancer cells, LNCaP, C4-2 and DUVIPR were stained with CD133 antibodies, and MCF7 breast cancer cells were double stained with CD44 and CD24 antibodies and analyzed by flow cytometry. CSCs (CD133^+^ population in prostate cancer cells, and CD44^+/high^CD24^−/low^ population in MCF7) are shown in magenta and the percentage of CSCs is indicated. (**b**) CSC enrichment by tumorosphere formation: cancer cells were grown in normal RPMI medium with 10% FBS (parental) or sphere medium for 8 days on ultra-low attachment plates. Phase-contrast images are shown. Formation of typical unattached floating spheres can be observed. Scale bar=100 *μ*m. (**c**) Individual sphere cells were stained with CD133 or CD44/CD24 antibodies and FAC-sorted. Pre-sort and post-sorted dot plots are shown. (**d**) Extended proliferative capacity of CSCs and spheres compared with parental cells: indicated cells were seeded at a density of 10^4^ in 96-well plates. After 3 days, the cells were incubated with 10 *μ*M BrdU, followed by anti-BrdU antibody-HRP. (**e**) Parental cells, sphere cells and sorted CSCs were seeded at one cell/well in 96-well plates and sphere-forming efficiency was compared. The number of spheres (>100 *μ*m) formed was counted under microscope. (**f**) Enhanced invasion capacity of CSCs and spheres: indicated cells were seeded, and allowed to invade towards bottom side of membrane containing growth factor supplements. After 24 h, fluorescence measurements were recorded according to Assay Instructions. (**g**) Increased migration capacity of CSCs and spheres: indicated cells were seeded, and allowed to migrate towards growth factor supplements for 24 h. Colorimetric measurements were taken according to Assay Instructions. (**h**) Western blot show that several stemness markers in CSCs and spheres as compared with parental cells were upregulated. *β*-Actin was used as loading control. The error bars represent S.D. of the biologic triplicates. All experiments presented in this figure are representative of at least three independent experiments

As CD133 glycoprotein is one of the most promising and well-investigated CSC markers of various cancer types,^31, 32, 33, 34^ we used CD133 for the identification of CSCs. Flow cytometry analysis of parental cancer (PC) cells stained with CD133 antibody revealed a very small subset of CD133^+^ cells (Figure 2a). However, we were unable to detect CD133^+^ population in MCF7 cells (not shown), an observation consistent with the previous findings.^35^ Several previous reports and our own experiments confirmed the existence of CD44^+/high^/CD24^−/low^ population in MCF7 cells, which are well demonstrated for their stemness potential.^12, 36^ Therefore, we used a combination of CD44/CD24 markers for obtaining CSCs from MCF7 cells, which accounted for about 1% (Figure 2a).

As quantification of such a very low number of CSCs in PC cells can make the assay sensitive to confounding effects, and are not sufficient to address our goals that demanded multiple experiments, we enriched CSCs by forming spheres. Individual PC cells at a density of 1–10 cells/*μ*l were cultured in sphere medium in ultra-low attachment plates. Under these suspension culture conditions, a few cells grew as freely floating non-adherent spheres (Figure 2b). Flow cytometry analysis of these sphere-derived cells (SC) with CD133 or CD44/CD24 antibodies revealed significantly enriched CSC population (Figure 2c).

To demonstrate whether these CD133^+^ and CD44^+/high^/CD24^−/low^ population can exhibit classical stem cell-associated features, we purified this population from spheres using FAC-sorting. The analysis of post-sorting cells clearly showed that the CD133^+^ or CD44^+/high^/CD24^−/low^ cells were purified to an extent of >90% (Figure 2c). Compared with PC, the SC and CSCs exhibited increased stem cell-associated features such as proliferation, sphere-forming ability, migration and invasion (Figures 2d–g). Furthermore, several stemness-associated transcription factors such as Oct4, Nanog, Gli-1 and Bmi as well as antiapoptotic BclXL and multidrug resistance protein ABCG2 were upregulated more in purified CSCs and moderately in SC (Figure 2h). We also found that the CD133^+^ or CD44^+/high^/CD24^−/low^ cells isolated directly from PC and from that of SC displayed similar stemness features (Supplementary Figure 1). Together, our results confirmed that the CD133^+^ population in prostate cancer cells and CD44^+/high^/CD24^−/low^ population in breast cancer MCF7 cells possessed typical stemness-associated characteristics, consistent with previous reports.^9, 32, 33, 34, 35, 36^ Thus, these cells, hereafter referred to as CSCs, were further used to understand the signaling mechanisms activated by neuropeptides.

### VIP protects CSCs from drug-induced apoptosis

**Figure 3:**
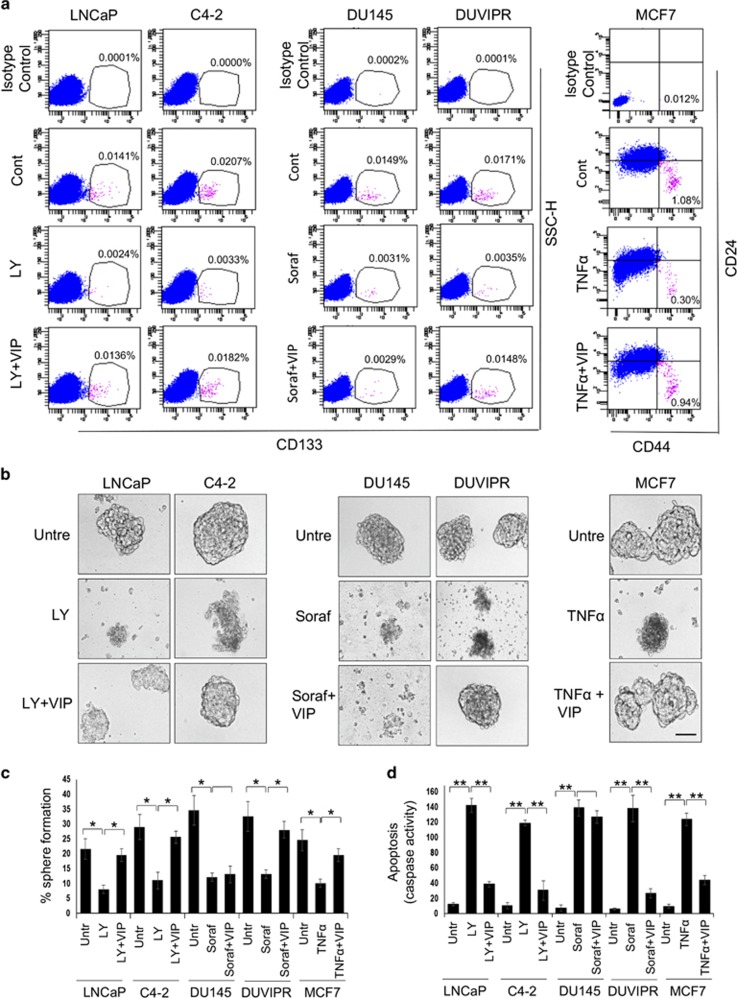
VIP protects spheres and purified CSCs from drug-induced apoptosis. (**a**) Indicated parental cells were starved for 12 h and apoptosis was induced by treating with either 20 *μ*M LY, 20 *μ*M sorafenib or 10 ng/ml TNF*α*. Fifteen minutes later, 100 nM of VIP was added. After 24 h, single cell suspensions were stained with CD133 antibodies, or double stained with CD44 and CD24 antibodies and analyzed by flow cytometry. Note that VIP restores the CSC population from drug-induced apoptosis. (**b**) Spheres derived from indicated cell lines were starved for 12 h and treated as in **a**. Phase-contrast images are shown. Scale bar=100 *μ*m. (**c**) Spheres in **b** were dissociated, and sphere-forming assay was performed by culturing cells in sphere medium for 8 days on ultra-low attachment plates. The number of reformed spheres was counted. (**d**) Purified CSCs were treated as in **a** and the caspase activity in the cell lysates was measured using the fluorogenic substrate Ac-DEVD-AMC. The *P*-values for the indicated comparisons were obtained by two-tailed independent Student’s *t*-test. *P<0.05, **P<0.01

As VIPR are abundantly expressed in cancer cells as well as tumors, and no information is available on the cytoprotective efficacy of VIP in CSCs, we tested whether VIP protects CSCs from apoptosis. Our previous findings suggest that while inhibition of PI3K/Akt with a pharmacological inhibitor LY294002 (LY) is sufficient to induce apoptosis in CSCs of LNCaP and C4-2 cells, TNF*α* can be cytotoxic to CSCs of MCF7 cells.^12^ However, inhibition of PI3K/Akt alone is not sufficient to induce apoptosis in CSCs of DU145 and DUVIPR as other survival kinases are also active. Thus, to induce apoptosis in CSCs of these cells, we used sorafenib, a multi-kinase inhibitor that can suppress action of tyrosine kinases and Ras-family kinases. Indeed, PI3K/Akt inhibitors, sorafenib and TNF*α* are widely used in clinic to treat patients with several cancer types.^37, 38, 39^ Parental cancer cells were treated with either of these anticancer drugs, followed by 100 nM of VIP for 24 h. This amount of VIP was reported to be physiologically relevant concentration.^40^ The cells were stained with CD133 or CD44/CD24 antibodies and analyzed by flow cytometry. There was a significant reduction in CSC population by treatment with drugs, but this frequency was restored by VIP (Figure 3a). As VIP alone could not enhance the frequency of CSCs either by conversion of PC to CSC or by inducing the proliferation of CSCs (Supplementary Figure 2), the observed effects of VIP are consistent with the idea that VIP protected CSCs from drug-induced cell death. Indeed, the observed cytoprotection was exclusively mediated by binding of VIP to the VIPR because CSCs of DU145 cells, which lack VIPR expression, were not rescued by VIP from apoptosis. Furthermore, culturing tumorospheres in media containing drugs resulted in apoptosis and subsequent disintegration of spheres, which was blocked by VIP (Figure 3b). Similarly, a significant decline in secondary sphere-forming capacity was observed in drug-treated cultures, but VIP could protect cells from apoptosis resulting in the increased number of secondary spheres (Figure 3c). To finally confirm the VIP-mediated CSC protection, we treated purified FAC-sorted CSCs with drugs followed by VIP, and apoptosis was measured by quantitating the caspase activity in the cell lysates. Drug-induced caspase activity was substantially reduced by treating CSCs with VIP (Figure 3d). Furthermore, compared with PC, the expression of VIP in CSC is very modest (Supplementary Figure 3). Taken together, our results confirm that VIP can protect CSCs from apoptosis induced by anticancer drugs.

### Cytoprotective efficacy of neuropeptides on CSCs

**Figure 4:**
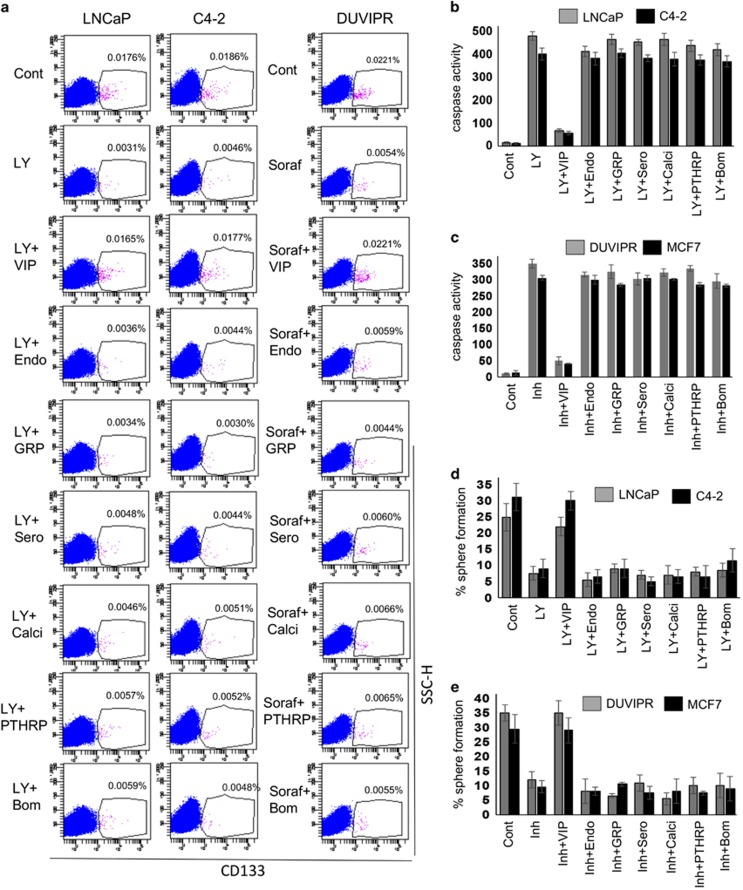
Cytoprotective efficiency of various neuropeptides on CSCs. (**a**) Parental cells were treated as in Figure 3a to induce apoptosis. Fifteen minutes later, the cells were treated with 100 nM of each of indicated neuropeptide. Single cell suspensions were stained with antibodies against CD133 and analyzed by flow cytometry. (**b** and **c**) Spheres derived from indicated cell lines were starved for 12 h and treated as in **a**. Caspase activity in the sphere cell lysates was measured as in Figure 3d. Inhibitor=Sorafenib for DUVIPR cells, and TNF*α* for MCF7 cells. (**d** and **e**) Spheres in **b** and **c** were dissociated, and sphere-forming assay was performed, and the number of reformed spheres was counted. Results suggest that antiapoptotic effect is specifically exhibited by VIP. Please see Supplementary Table 1 for *P*-values

We next investigated whether other neuropeptides can protect CSCs from apoptosis. Parental cancer cells were treated with drugs, followed by 100 nM each of neuropeptide or Sero. The cells were stained with CD133 antibodies and analyzed by flow cytometry. Except VIP, none of the other neuropeptides could restore the CSC frequency (Figure 4a). Similarly, the disintegration of spheres induced by drugs was not blocked by any of the tested neuropeptides except VIP (data not shown). Analysis of caspase activity in the cell lysates of tumorospheres revealed that only VIP provided the protection but not other neuropeptides (Figures 4b and c). Although there was a 5- to 6-fold reduction in the self-renewal ability of drug-treated sphere cells compared with control, neuropeptides other than VIP had no effect on the self-renewal ability (Figures 4d and e). Collectively, our results show that the cytoprotective mechanisms are activated exclusively by VIP.

### VIP-induced cytoprotection correlates with BAD phosphorylation in CSCs

**Figure 5:**
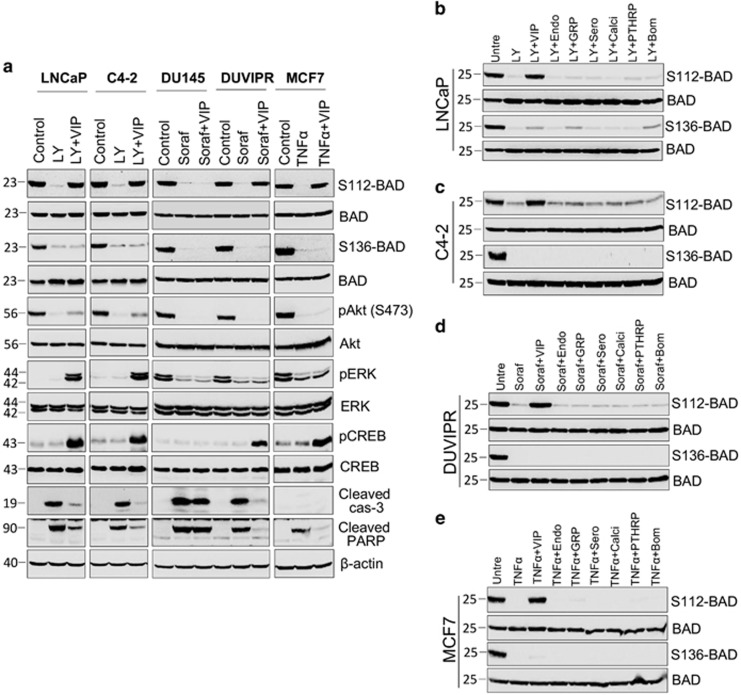
VIP induces BAD phosphorylation in CSCs. (**a**) CSCs were purified by FAC-sorting from spheres expressing HA-BAD. CSCs were placed in supplement-free basal DMEM medium for 3 h and treated with indicated inhibitors. Fifteen minutes after addition of inhibitors, 100 nM VIP was added. Three hours later, HA-BAD was immunoprecipitated from whole-cell lysates using HA antibodies (12CA5), and immunoblotted with indicated antibodies. Both S112-BAD and S136-BAD blots were stripped and reprobed with total BAD antibodies. Blots of phospho-Akt, phospho-ERK and phospho-CREB were, respectively, stripped and reprobed with Akt, ERK and CREB. Cleavage products of PARP and caspase-3 were used as apoptotic markers in whole-cell lysates. Note that MCF7 cells lack caspase-3 expression. (**b**–**e**): Spheres expressing HA-BAD were treated as indicated and BAD phosphorylation was detected as in **a**. Note that except VIP, no other neuropeptide induces BAD phosphorylation in tested CSCs. Both S112-BAD and S136-BAD blots were stripped and reprobed with total BAD antibodies

Previously, we showed that BAD has a key role in cytoprotection of DC cells and CSCs.^9, 10, 11, 12^ BAD can be phosphorylated at least on three different serine residues, S112, S136 and S155, that correlate with anti-apoptosis. However, it is not known whether VIP-induced protection is controlled by BAD in CSCs. To address this, we ectopically expressed hemagglutinin tagged BAD (HA-BAD) in spheres using lentiviral vector and CSCs were purified. Because commercially available antibodies are not sensitive enough to recognize endogenous S136-BAD phosphorylation, we performed initial experiments with ectopically expressed HA-BAD. CSCs expressing HA-BAD were treated with drugs followed by VIP and BAD phosphorylation was detected on immunoprecipitated HA-BAD. Inherently, BAD was kept phosphorylated at S112 and S136 in control CSCs, but was dephosphorylated at both sites by drugs (Figure 5a). However, VIP could induce BAD phosphorylation specifically at S112 on both endogenous and overexpressed BAD (Supplementary Figure 4). As VIP restored only S112 phosphorylation that could be detected on endogenous BAD, the rest of the experiments were performed on endogenous BAD. We also found a correlation between BAD phosphorylation and survival as VIP inhibited the cleavage of PARP and caspase-3 (Figure 5a). Together, our results show an excellent correlation between drug-induced apoptosis and BAD dephosphorylation, and between VIP-induced survival and BAD phosphorylation in CSCs.

### Other neuropeptides do not induce BAD phosphorylation

Our finding showing VIP-induced survival correlated with BAD phosphorylation prompted us to investigate whether other neuropeptides, which could not protect CSCs from apoptosis, induce BAD phosphorylation or not. Tumorospheres expressing HA-BAD were treated with drugs and 100 nM of neuropeptide or Sero and BAD phosphorylation was evaluated. None of the neuropeptides, except VIP, restored BAD phosphorylation, confirming that cytoprotection and BAD phosphorylation are exclusively triggered by VIP (Figures 5b–e).

### VIP-induced signaling pathways in CSCs

Next, we focused on identification of signaling pathways activated by VIP. VIP has been shown to activate multiple pathways such as EGFR, ERK, Akt, Protein Kinase A (PKA) and Src kinase.^9, 41, 42, 43^ To delineate which of these pathways are activated by VIP that control cytoprotection and BAD phosphorylation in CSCs, we measured the activation of Akt, Ras/MAPK and PKA. When activated, Akt is phosphorylated at Ser473 and ERK1/2 are phosphorylated at Thr202/Tyr204. CREB is a substrate of PKA, which is phosphorylated at Ser133 by activated PKA. Thus, to judge the activation of PKA, antibodies that recognize CREB phosphorylation are widely used. Both ERK1/2 and PKA were significantly activated by VIP in CSCs of LNCaP and C4-2. In contrast, only PKA was activated in CSCs of DUVIPR and MCF7. However, Akt was not activated by VIP in any of the CSCs (Figure 5a).

**Figure 6:**
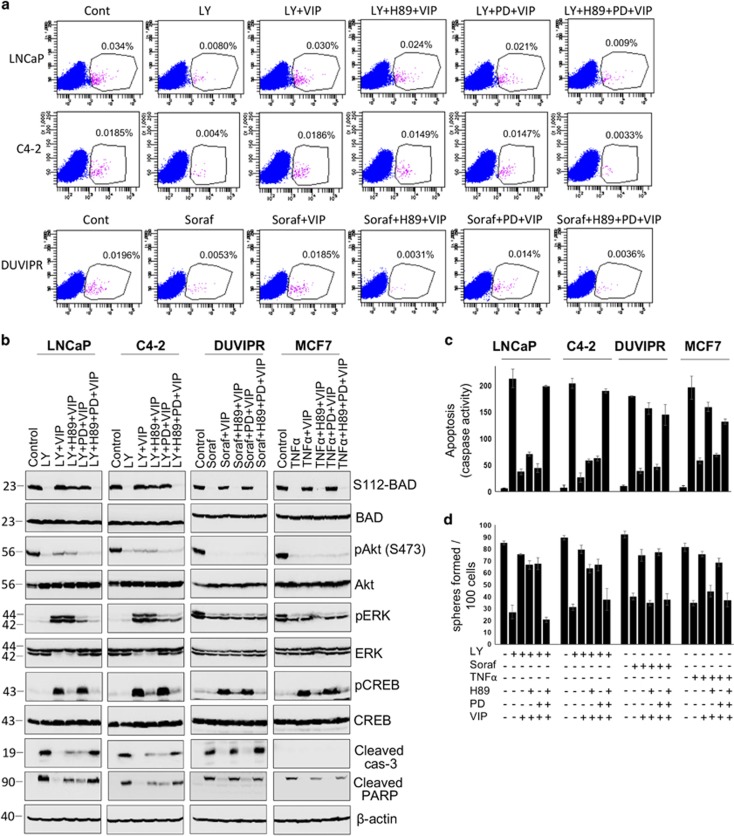
VIP-induced cytoprotective mechanisms in CSCs. (**a**) Parental cells were treated with either 20 *μ*M LY, 20 *μ*M sorafenib or 10 ng/ml TNF*α* and 50 *μ*M PD98059 or 10 *μ*M H89. Fifteen minutes later, the cells were treated with 100 nM of VIP. Single cell suspensions were stained with antibodies against CD133 and analyzed by flow cytometry. (**b**) Sorted CSC were placed in supplement-free basal DMEM medium for 12 h and treated with indicated inhibitors, and VIP was added 15 min later. Cell lysates were immunoblotted with indicated antibodies as in Figure 5a. Blots of S112-BAD, phospho-Akt, phospho-ERK and phospho-CREB were, respectively, stripped and reprobed with total BAD, Akt, ERK and CREB. Cleavage products of PARP and caspase-3 were used as apoptotic markers in whole-cell lysates. (**c**) Caspase activity in cell lysates of **b** was measured as described in Figure 3d. (**d**) Sphere-forming assay was performed using cells in **b**, and the number of spheres generated was counted. The error bars represent S.D. of the biologic triplicates. All experiments presented in this figure are representative of three independent experiments. Please see Supplementary Table 2 for *P*-values

**Figure 7:**
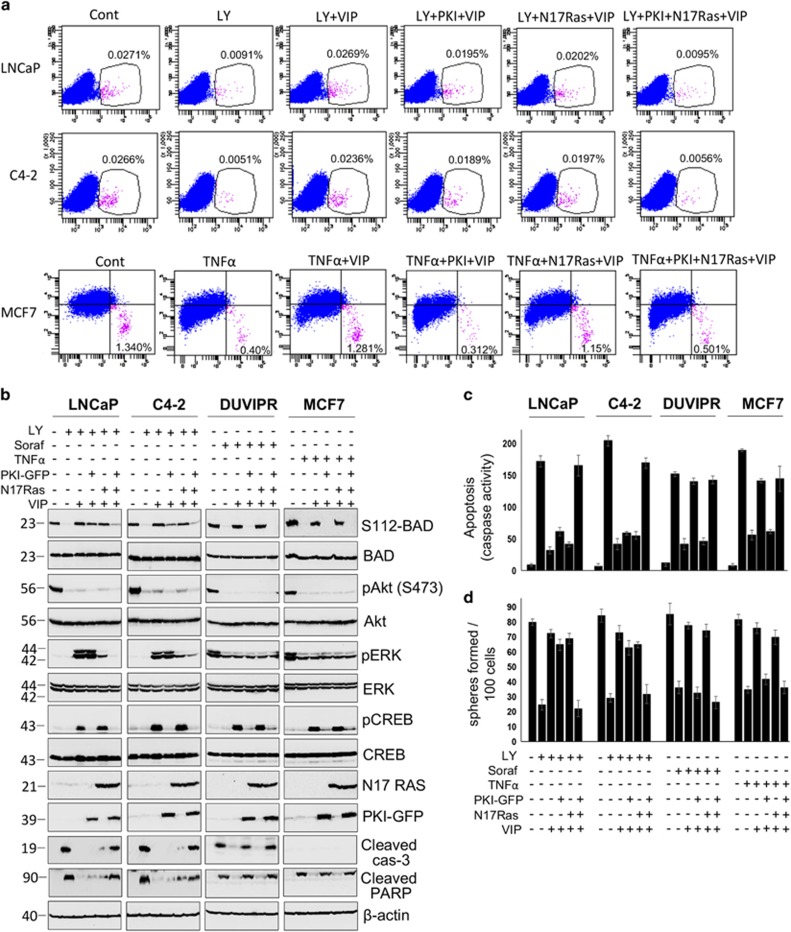
VIP-induced cytoprotection is abrogated by dominant-negative PKI-GFP and N17 Ras. (**a**) Parental cells expressing either empty vector or PKI-GFP and/or HA-N17 Ras were treated as in Figure 6a. Individual cells were stained with antibodies against CD133 or CD44/CD24 and analyzed by flow cytometry. (**b**) Purified CSCs, expressing PKI-GFP and/or HA-N17 RAS were treated with indicated inhibitors. VIP was added 15 min later. BAD phosphorylation was detected as in Figure 5a. Blots of S112-BAD, phospho-Akt, phospho-ERK and phospho-CREB were, respectively, stripped and reprobed with total BAD, Akt, ERK and CREB. Expression of PKI-GFP and HA-N17 Ras were, respectively, detected using antibodies against GFP and HA. Cleavage products of PARP and caspase-3 were used as apoptotic markers in whole-cell lysates. (**c**) Caspase activity in cell lysates of **b** was measured as described in Figure 3d. (**d**) Sphere-forming assay was performed using cells in **b**, and the number of spheres generated was counted. The error bars represent S.D. of the biologic triplicates. All experiments presented in this figure are representative of two independent experiments. Please see Supplementary Table 3 for *P*-values

As there was a strong correlation between VIP-induced BAD phosphorylation and activation of MAPK and PKA, we further analyzed these pathways in greater detail. PD98059 (PD) and H89 are widely used specific inhibitors of MEK1/2 and PKA, respectively. Purified CSCs were treated with anticancer drugs as well as PD, H89 or both. VIP was added 15 min after the addition of drugs. Western blot analysis showed that while PD and H89 could block the activation of MAPK and PKA, respectively, the cytoprotection and BAD phosphorylation induced by VIP were only partially decreased by each of these inhibitors in CSCs of LNCaP and C4-2 (Figures 6a–c). Simultaneous inhibition of both MAPK and PKA with a combination of PD and H89 led to significant suppression of cytoprotection and BAD phosphorylation. However, in CSCs of DUVIPR and MCF7, inhibition of PKA alone could abrogate BAD phosphorylation and cytoprotective effect of VIP. Similarly, although there was a substantial reduction in the number of spheres formed only when cells were treated with a combination of PD and H89 in LNCaP and C4-2 cells, PKA inhibition alone reduced the sphere-forming capacity of DUVIPR and MCF7 (Figure 6d). Similar results were obtained when activity of Ras and PKA were inhibited by dominant-negative N17 Ras and PKI-GFP, respectively, in CSCs (Figure 7). In summary, VIP-induced BAD phosphorylation and cytoprotection are mediated via MAPK and PKA pathways in CSCs of LNCaP and C4-2, whereas in DUVIPR and MCF7, these events are signaled through PKA pathway only. Activation of these pathways alone is sufficient to protect from drug-induced cell death.

### Absence of BAD desensitizes CSCs from drug-induced apoptosis

**Figure 8:**
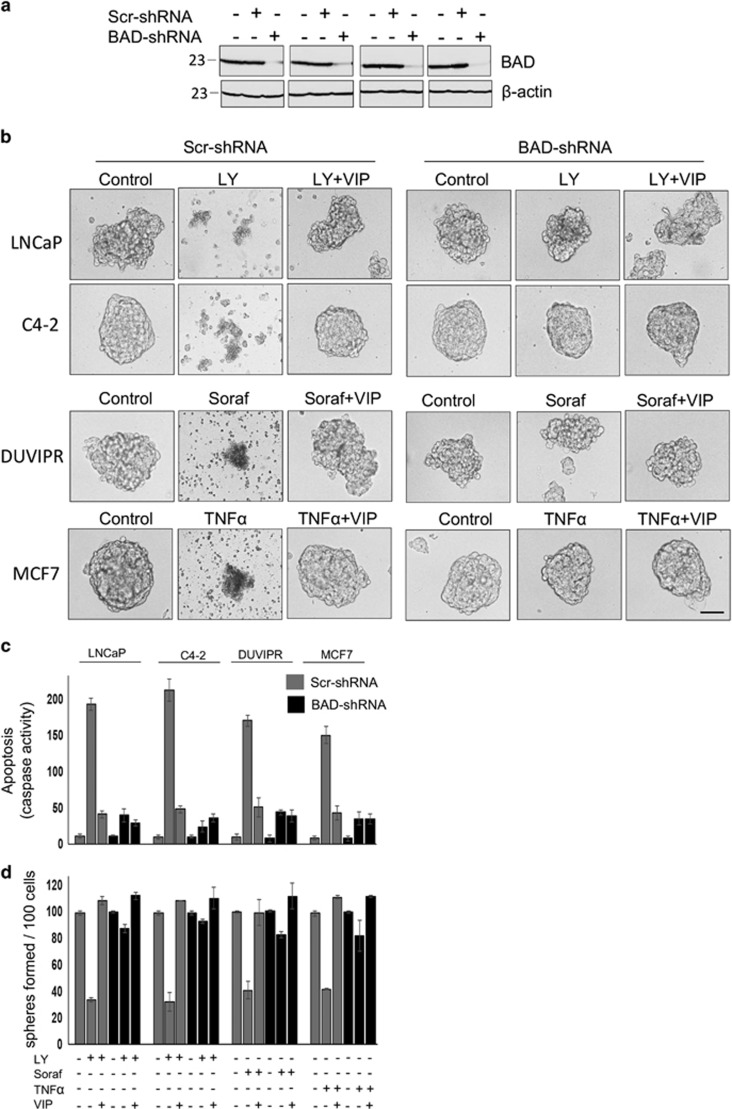
Absence of BAD desensitizes CSCs from drug-induced apoptosis. (**a**) Spheres were produced from parental cells infected with lentiviral vectors containing BAD-shRNA or scrambled-shRNA. Sphere cell lysates were collected after 8 days, and immunoblotted with antibodies against BAD. Equal loading was judged by *β*-actin. Note that BAD expression was substantially suppressed by BAD-shRNA. (**b**) Tumorospheres expressing BAD-shRNA or Scr-shRNA were incubated for 24 h with indicated inhibitors and VIP in supplement-free DMEM medium. Phase-contrast images of spheres are shown. Scale bar=100 *μ*m. (**c**) Purified CSCs expressing either BAD-shRNA or Scr-shRNA were treated as in **b**, and the caspase activity was measured form whole-cell lysates. (**d**) Sphere-forming assay was performed using 100 cells in **c**, and the number of spheres formed was counted. The error bars represent S.D. of the biologic triplicates. All experiments presented in this figure are representative of three independent experiments. Please see Supplementary Table 4 for *P*-values

To test whether BAD expression is necessary for drug-induced apoptosis in CSCs, we infected spheres with well-characterized lentiviral vector containing either scr-shRNA or BAD-specific shRNA^9, 12^ that greatly reduced BAD expression (Figure 8a). Although anticancer drugs could induce apoptosis in scr-shRNA-spheres and purified CSCs, none of these drugs could induce apoptosis in BAD knockdown cells (Figures 8b and c). Similarly, anticancer drugs could not reduce the sphere-forming ability of CSCs devoid of BAD expression (Figure 8d). Thus, BAD expression is essential for induction of apoptosis in CSCs.

### BAD phosphorylation is essential for VIP-induced cytoprotection in CSCs

**Figure 9:**
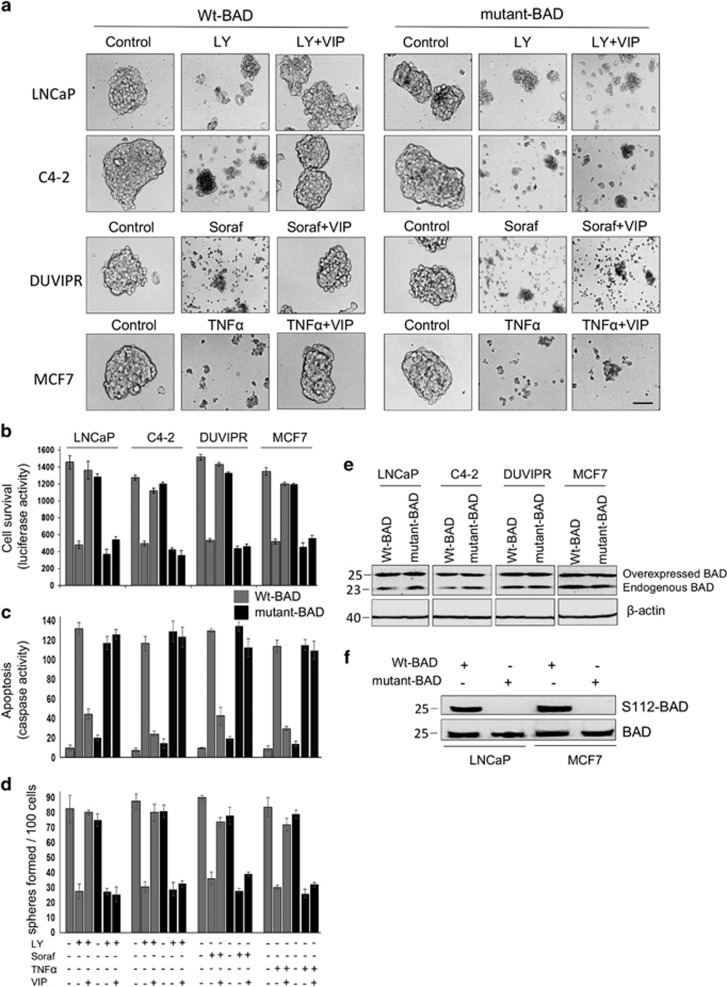
BAD phosphorylation is essential for VIP-induced cytoprotection in CSCs. (**a**) Tumorospheres were infected with lentiviral vectors expressing wt-BAD or mutant-BAD. After 24 h, spheres were placed in supplement-free DMEM containing indicated inhibitors and VIP for 3 h. Phase-contrast images of spheres are shown. Scale bar=100 *μ*m. (**b**) CSCs were purified by FAC-sorting from spheres that express wt-BAD or mutant-BAD and luciferase. CSCs were placed in supplement-free DMEM containing indicated inhibitors and VIP for 3 h. Luciferase activity was measured from cell lysates to judge the CSC survival. (**c**) Caspase activity was measured from whole-cell lysates as collected in **b**. (**d**) Sphere-forming assay was performed using 100 cells in **b**, and the number of spheres formed was counted. (**e**) Expression levels of wt-BAD and mutant-BAD are shown. (**f**) Cell lysates in **a** were probed with indicated antibodies. Note that mutant-BAD behaves like dephosphorylated BAD as S112-BAD antibodies do not recognize phosphorylation on S112 of mutant-BAD. The error bars represent S.D. of the biologic triplicates. All experiments presented in this figure are representative of three independent experiments

To determine the role of BAD phosphorylation in the VIP-induced antiapoptotic effect, we expressed wt-BAD or a mutant-BAD in which Ser was replaced with Ala (S112A) and thus cannot be phosphorylated. Although VIP protected wt-BAD-expressing spheres from drug-induced apoptosis, there was a robust apoptosis and subsequent disintegration of spheres even after treatment of mutant-BAD-expressing spheres with VIP (Figure 9a). Similar results were noticed using cell survival assay (Figure 9b). To further confirm these results, we purified CSCs from spheres and apoptosis was quantified. Compared to wt-BAD-expressing CSCs, the antiapoptotic effect of VIP was significantly reduced in mutant-BAD-expressing CSCs (Figure 9c). In a self-renewal assay, VIP could not enhance the sphere-forming capacity of CSCs expressing mutant-BAD (Figure 9d). Figure 9e shows that both wt-BAD and mutant-BAD were expressed at similar levels in cells, and Figure 9f shows that the mutant-BAD behaves like dephosphorylated BAD. Thus, the protection of CSCs by VIP is predominately mediated by S112-BAD phosphorylation.

## Discussion

In this paper, we focused on understanding the antiapoptotic roles of several neuropeptides in CSCs. We show that, except VIP, none of the other neuropeptides could protect CSCs from drug-induced apoptosis. Specifically, the antiapoptotic effect of VIP is mediated by VIPR- and PKA/MAPK-dependent BAD phosphorylation. As far as we know, this is the first report that comprehensively investigates the cytoprotective effects of neuropeptides in CSCs.

The major limitation to use cell line-derived CSCs is that these cells are adapted to the *in vitro* culture conditions, so they may not fully resemble their counterparts in primary tumors. However, because of limited availability of patient specimens, the use of cell lines is still a viable option for obtaining enough CSCs to address the signaling mechanisms. Therefore, we relied on the cell lines for isolation of CSCs, which were further enriched by sphere formation. These spheres and CSCs exhibited typical stem cell-associated features and upregulated several stemness markers.

Drug resistance is a complicated process that involves diverse mechanisms. It has been shown that instead of eliminating CSCs, chemotherapeutic drugs have enriched CSC population.^1, 2, 3, 4^ Thus, targeting and elimination of CSCs is one of the best strategies to treat cancer. Resistance to chemotherapy frequently results from cell extrinsic factors derived from the tumor microenvironment (TME) or niche. Many tumor types involve CSCs in the TME milieu.^44^ A wide variety of cytokines, chemokines, growth factors, neuropeptides and proteinases, which are produced in the niche, dramatically limits the effective delivery of anticancer drugs to tumor/CSC foci, and afford drug resistance.^45, 46^

Various neuropeptides are produced in the TME, among which VIP deserves a special emphasis. Neuroendocrine (NE) differentiation of tumor cells correlates with unfavorable clinical outcome, and VIP can induce NE differentiation.^42^ NE tumors are difficult to treat as they display high resistance to apoptosis and the quantity of NE cells in a tumor correlates with poor prognosis.^47^ VIPomas arising from several organs are a special type of neuroendocrine tumors that secrete massive amounts of VIP.^48, 49^ Overexpression of VIP and its receptors in various neoplasia has been found.^25, 26, 27, 28, 29^ VIP is also able to induce malignant transformation of human prostate epithelial cells by epithelial mesenchymal transition.^50^ Thus, because of its diverse roles in carcinogenesis and metastasis, we focused on unveiling the cytoprotective role of VIP in CSCs.

Activation of PI3K/Akt and Ras/MAPK pathways is implicated in the therapeutic resistance of CSCs.^13, 51, 52^ We previously showed that EGF and estradiol induced PI3K/Akt and MAPK pathways to protect CSCs from drug-induced apoptosis.^12^ Our present results show that among several neuropeptides the cytoprotection is displayed exclusively by VIP. However, we cannot rule out the potential role of other neuropeptides in the biology of CSCs. For example, it has been recently shown that the CD133^+^ cells of small cell lung cancer expressed elevated levels of GRP, and a novel neuropeptide antagonist, peptide-1, is able to inhibit the growth of these cells.^53^ Therefore, it remains to be investigated the diverse roles of neuropeptides in various aspects of CSC’s biology.

PKA pathway seems to be a predominant antiapoptotic pathway activated by VIP in CSCs. PKA pathway is aberrantly regulated in different cancers and promotes tumor growth. While the regulatory subunit of PKA, RI*α*, is upregulated in several tumors, inhibition of RI*α* expression resulted in growth arrest of several tumor cell lines.^54^ Furthermore, PKA has been shown to be involved in drug resistance. In breast cancer, activation of PKA signaling conferred trastuzumab resistance.^55^ However, activation of PKA signaling to confer drug resistance in CSCs has not been reported earlier. Thus, our results show that VIP provides another layer of drug resistance by inducing activation of PKA in CSCs of breast and prostate cancer.

Many cancer cells activate redundant signals that act as bypass or backup signaling, thereby fostering adaptation to drug inhibition. This study highlights the role of MAPK signaling as a critical backup system in CSCs. Under sustained suppression of PKA signaling, VIP could re-wire survival signaling pathways by inducing MAPK activation that serves as a bypass pathway to confer drug resistance. This will have therapeutic implications. Targeting either of these pathways alone can become ineffective because another parallel pathway supports CSC’s survival. In this case, these two pathways develop a synthetic lethal relationship where the inactivation of one of these pathways would be supported by the other pathway, and for the most effective treatment, both pathways need to be targeted. However, these *in vitro* observations need to be confirmed in an appropriate animal model or clinical samples.

Although myriad of antiapoptotic pathways are activated by diverse growth factors, cytokines and neuropeptides, we showed that they are integrated into a few survival kinases positioned downstream of these pathways to regulate cell survival.^9, 10, 11, 12^ P90RSK, BMK1/ERK5, Akt, PAK1, PKA and p70S6 kinase are some of the well-established kinases, and interestingly, BAD is a direct substrate of these kinases.^9, 10, 11, 12, 16, 17, 18^ Analysis of cytoprotection and BAD phosphorylation in CSCs demonstrated that VIP-induced antiapoptotic signaling is predominately dependent on BAD phosphorylation at S112. Both PKA and MAPK pathways controlled BAD phosphorylation exclusively at S112, and the cytoprotective effect of VIP is reduced in CSCs that expressed a mutant-BADS112A compared with those expressing wt-BAD, highlighting the key role of BAD in survival of CSCs. Although PKA can directly phosphorylate BAD, the BAD kinase activated by Ras/MAPK pathway remains to be identified in CSCs. Although it is well known that CSCs can give rise to more differentiated cancer cells that form the bulk of the tumor, recent findings suggest that the DC cells can switch to CSC phenotype under appropriate conditions.^56^ Thus, targeting both DC cells and CSCs is a more appropriate strategy to treat cancer. In this regard, our present results and previous one^9, 10, 11, 12^ suggest that BAD is one of such promising targets because it controls survival and drug resistance in both DC cells and CSCs.

Our findings showing higher expression of VIPR in tumor tissue than in normal tissue, and that VIPR mediate drug resistance in CSCs point to VIPR also as a promising target. This has significant implications in diagnostics and treatment. In addition to targeting the VIP-induced signaling pathways to minimize the drug resistance, cytotoxic drugs conjugated to VIP could be more effectively targeted to the tumor sites and eliminate both CSCs and DC. Such trails as VIP-guided nanotherapy are already underway.^57^ Similarly, VIPR analogs were labeled with ^64^Cu and successfully utilized in positron emission tomography imaging of breast cancers both *in vitro* and *in vivo*.^58^

Taken together, our results suggest that protection from apoptosis by VIP provides yet another mechanism that contributes to resistance of CSCs to anticancer drugs. These results might have translational implications for the effective treatment of prostate and breast cancers, and may be potentially other malignancies. Further experiments are required to prove our preliminary observations in appropriate animal models to determine the role of VIP-induced cytoprotection of CSCs in tumors.

## Materials and methods

### Cell lines and reagents

LNCaP, DU145 and MCF7 cells were procured from American Type Collection Centre (Manassas, VA, USA). C4-2 cell line was a gift from Leland Chung, Emory University (Atlanta, GA, USA). DUVIPR cells were generated by introducing VIPR through lentiviral vector. Transformed cells were selected in puromycin antibiotic and the individual colonies were screened for the expression of VIPR. Unless specified, all antibodies were obtained from Cell Signaling Technology (Beverly, MA, USA), and all chemicals were purchased from Sigma (Milwaukee, WI, USA). VIPR antibody was from Millipore (Chicago, IL, USA). LY294002, VIP, PD98059, Z-VAD-FMK, Protein G-agarose beads, DEVD-7-amino-methyl-coumarin (Ac-DEVD-AFC) were from Calbiochem (Chicago, IL, USA). TNF*α* was from Peprotech (Rock Hill, NJ, USA).

### Cell culture

All cell lines were cultured in RPMI-1640 medium (Invitrogen, Grand Island, NY, USA) supplemented with 10% fetal bovine serum and 1% penicillin/streptomycin in a humidified 5% CO_2_ incubator maintained at 37 °C.

### Patient specimen

Breast tumor tissue and corresponding healthy breast tissue were obtained from the Weill Cornell Medicine-Qatar Genetic Medicine Database and the Arab Breast and Prostate Cancer Consortium Bio-repository, as described.^12^ DNA, RNA and protein were extracted from these tissues using AllPrep DNA/RNA/Protein Kit (Qiagen, Hilden, Germany). VIPR1 expression at RNA level was quantified by standard quantitative real-time PCR and at protein level by western blot.

### Sphere culture

Individual cells at 1–10 cells/*μ*l were plated on low attachment plates with ultra-low attachment surfaces (Fischer Scientific Co., Pittsburgh, PA, USA) in sphere medium consisting of serum-free DMEM-F12 (1:1) supplemented with 20 ng/ml EGF, 10 ng/ml basic-FGF, 5 *μ*g/ml insulin, 1% N2 supplement, 1X B27 (both from Invitrogen) and 1% penicillin/streptomycin. Fresh growth factors were added to sphere culture every third day. The spheres were collected after 7–10 days and either trypsinized for flow cytometry analysis or protein extracted for western blotting. Where required, sphere number was counted manually using microscope.

### Flow cytometry analysis, western blot and immunoprecipitation

Spheres or monolayer cultures were trypsinized, washed in staining solution containing Ca^2+^- and Mg^2+^-free PBS with 1 mM EDTA, 25 mM HEPES and 0.5% FBS. Single cell suspensions were incubated with CD133-PE or CD44-PE/CD24-APC antibodies (BD Biosciences, San Jose, CA, USA) for 30 min on ice. The cells were rinsed twice in staining solution, and analyzed by FACS LSRFortessa Analyzer (BD Biosciences). To purify CSCs, stained cells were FAC-sorted using FACSAria sorter (BD Biosciences). Western blotting and immunoprecipitations were performed as described earlier.^9, 10, 11, 12^

### Proliferation, migration and invasion assays

BrdU cell proliferation assay kit (Cell Signaling), Colorimetric Cell Migration Assay kit (QCM 24-well, Millipore), and Fluorometric Cell Invasion kit (QCM 24-well, Millipore) were used to measure proliferation, migration and invasion capacity of parental cells, spheres and purified CSC, respectively, using Assay Instructions.

### Apoptosis and survival assays

From spheres: lentiviral production and transduction were performed according to the recommendations of the manufacturer (LentiX system from Clontech, Mountain View, CA, USA). Parental cells were cultured in sphere medium for 8 days. Where required, CSCs were purified from these spheres by FAC-sorting. Spheres or purified CSCs were starved for 12 h and treated with inhibitors and VIP. Twenty-four hours after addition of inhibitors, spheres or CSCs were either used for generation of secondary spheres, or lysed in cell lysis buffer. Apoptosis was quantified from cell lysates by measuring caspase-3,7 activity with the Ac-DEVD-AFC as described.^12^ Another part of the lysate was used in western blotting. In some experiments, spheres at day 5 were co-infected with lentiviral vectors containing either wt-BAD or mutant-BAD and luciferase. On day 7, CSCs were purified from these spheres if required. Spheres or purified CSCs were placed in supplement-free basal DMEM medium for 3 h, and then treated with inhibitors and VIP. Three hours after addition of inhibitors, spheres were either used for generation of secondary spheres or lysed in cell lysis buffer. This whole-cell lysate was used to measure apoptosis or quantify the cell survival by measuring the luciferase activity using a kit from Promega (Madison, WI, USA) and for western blotting.

### BAD knockdown by RNA interference

Parental cells were infected with PLL3.7 lentiviral vector containing either shRNA specific to BAD or a scramble-shRNA as reported by us.^9^ Three days post infection, the cells were subjected to sphere formation. After 7–8 days, either spheres were treated with inhibitors or CSCs were purified and apoptosis assays were performed.

### Statistical analysis

Paired Student’s *t*-test was used to compare differences in BAD expression between normal and tumor tissues. A *P*-value <0.05 was considered significant. All the statistical tests were two-sided. Unless otherwise stated, the error bars represent S.D. of the biologic triplicates.
