# Supplementary figures and images for: Cytoprotective effect of neuropeptides on cancer stem cells: vasoactive intestinal peptide-induced antiapoptotic signaling
Source: Cell Death Dis. 2017 Jun 1;8(6):e2844–. doi: 10.1038/cddis.2017.226 (PMC5520887; doi:10.1038/cddis.2017.226)

Suppl. Fig-1:

## Sphere formation

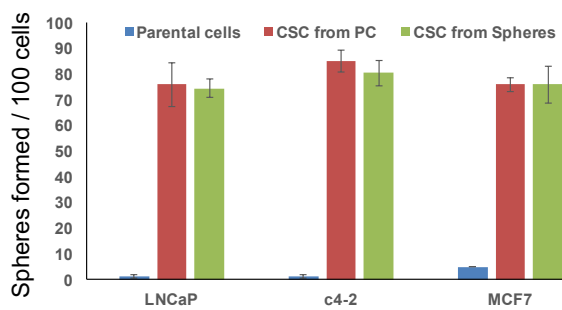

## Invasion capacity

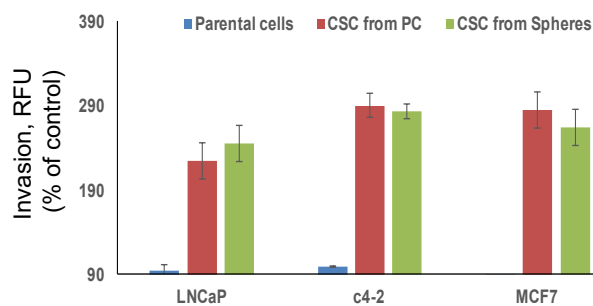

Suppl. Fig-2:

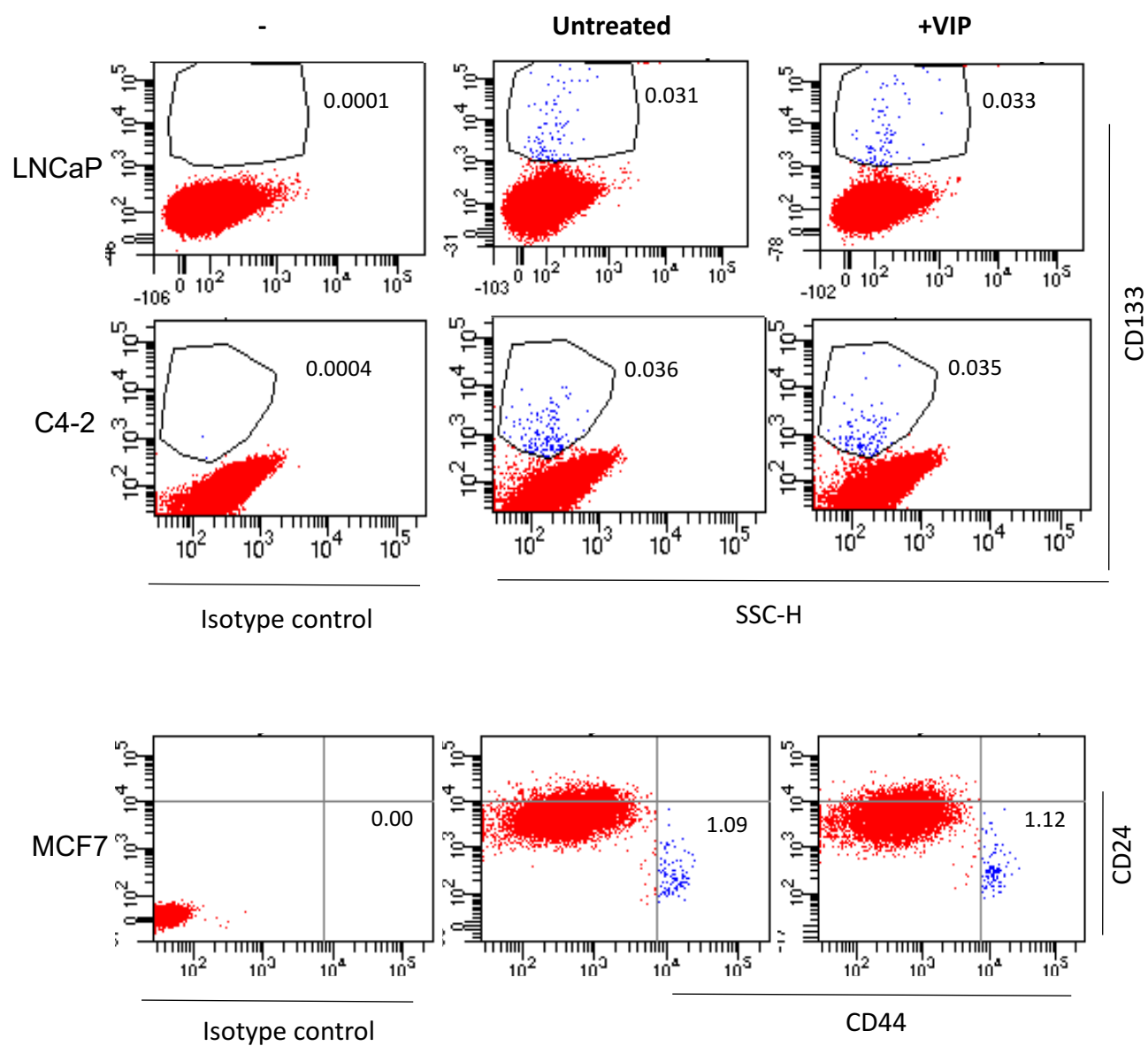

Suppl. Fig-3:

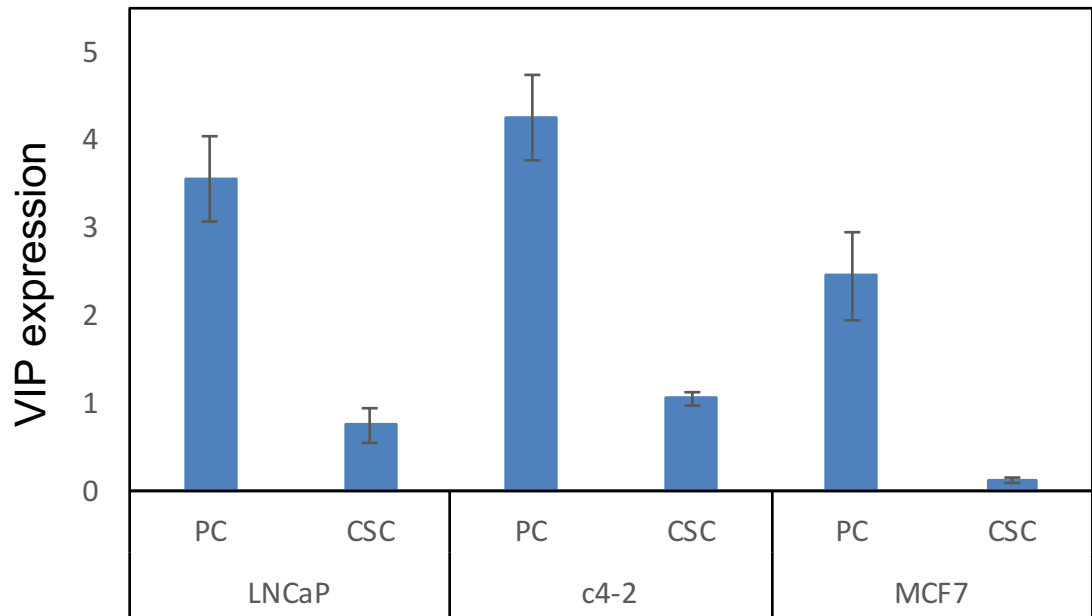

Suppl. Fig-4:

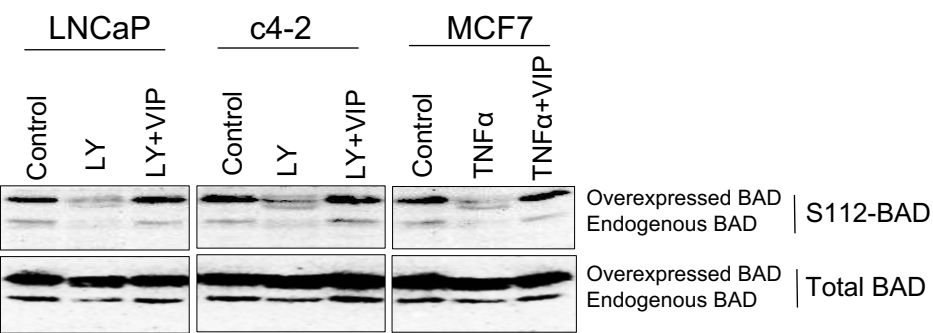

Supplement: Supplementary Figures [file cddis2017226x1.pdf]
