# Supplementary material for: Cytoprotective effect of neuropeptides on cancer stem cells: vasoactive intestinal peptide-induced antiapoptotic signaling
Source: Cell Death Dis. 2017 Jun 1;8(6):e2844–. doi: 10.1038/cddis.2017.226 (PMC5520887; doi:10.1038/cddis.2017.226)
Supplement: Supplementary Tables [file cddis2017226x2.pdf]

Supplementary Table-1: p values for various comparisons of caspase activity and sphere formation: Cytoprotective effects of various neuropeptides on CSCs.

Treatment	LNCaP		C4-2		DUVIPR		MCF7	
	Caspase activity	Sphere formation	Caspase activity	Sphere formation	Caspase activity	Sphere formation	Caspase activity	Sphere formation
Control vs Inh	***	*	**	*	***	*	***	*
Inh vs Inh+VIP	**	*	**	*	**	*	***	*
Inh vs Inh+Endo								
Inh vs Inh+GRP								
Inh vs Inh+Sero								
Inh vs Inh+Calci								
Inh vs Inh+PTHRP								
Inh vs Inh+Bom								

The *p* values for the indicated comparisons were obtained by two-tailed independent Student's *t*-test. *p<0.05, **p<0.01, ***p<0.001. Only the significant ones are marked.

Supplementary Table-2: p values for various comparisons of caspase activity and sphere formation: VIP-induced cytoprotective mechanisms in CSCs

Treatment	LNCaP		C4-2		DUVIPR		MCF7	
	Caspase activity	Sphere formation	Caspase activity	Sphere formation	Caspase activity	Sphere formation	Caspase activity	Sphere formation
Control vs Inh	**	**	**	**	***	**	**	**
Inh vs Inh+VIP	**	**	**	**	***	*	**	**
Inh vs H89+VIP	**	*	**	**				
Inh vs PD+VIP	**	*	**	*	***	**	*	**
Inh vs H89+PD+VIP								

The *p* values for the indicated comparisons were obtained by two-tailed independent Student's *t*-test. *p<0.05, **p<0.01, ***p<0.001. Only the significant ones are marked.

Supplementary Table-3: p values for various comparisons of caspase activity and sphere formation: VIP-induced cytoprotection is abrogated by dominant negative PKI-GFP and N17Ras

**Treatment**	**LNCaP**		**C4-2**		**DUVIPR**		**MCF7**	
	Caspase activity	Sphere formation	Caspase activity	Sphere formation	Caspase activity	Sphere formation	Caspase activity	Sphere formation
Control vs Inh	**	**	***	**	***	*	***	**
Inh vs Inh+VIP	**	**	**	**	**	**	**	**
Inh vs H89+VIP	**	**	**	*			**	
Inh vs PD+VIP	**	**	**	**	**	*	***	*
Inh vs H89+PD+VIP								

The *p* values for the indicated comparisons were obtained by two-tailed independent Student's *t*-test. *p<0.05, **p<0.01, ***p<0.001. Only the significant ones are marked.

Supplementary Table-4: p values for various comparisons of caspase activity and sphere formation: Absence of BAD desensitizes CSCs from drug-induced apoptosis

**Treatment**	**LNCaP**		**C4-2**		**DUVIPR**		**MCF7**	
	Caspase activity	Sphere formation	Caspase activity	Sphere formation	Caspase activity	Sphere formation	Caspase activity	Sphere formation
Inh (Scr shRNA) vs Inh (BADshRNA)	**	**	**	**	**	*	**	*
Inh+VIP (Scr shRNA) vs Inh+VIP(BADshRNA)								

The *p* values for the indicated comparisons were obtained by two-tailed independent Student's *t*-test. *p<0.05, **p<0.01. Only the significant ones are marked.
