# Supplementary material for: Cytoprotective effect of neuropeptides on cancer stem cells: vasoactive intestinal peptide-induced antiapoptotic signaling
Source: Cell Death Dis. 2017 Jun 1;8(6):e2844–. doi: 10.1038/cddis.2017.226 (PMC5520887; doi:10.1038/cddis.2017.226)
Supplement: Supplementary Figures and Tables [file cddis2017226x3.docx]

**Supplementary Figures:**

**Supplementary Figure-1:** CSCs purified directly from parental cells and from spheres exhibit similar stem cell-associated characteristics: **(A)** Indicated cells were seeded at 1 cell/well in 96-well plate and sphere-forming efficiency was compared. The number of spheres (>100 μm) formed was counted under microscope. Note that CSC purified directly from parental cells and CSC purified from spheres exhibited comparable invasive and sphere forming capacity. **(B)** Parental cells, CSCs purified directly from parental cells and CSCs purified from spheres were seeded, and allowed to invade towards bottom side of membrane containing growth factor supplements. After 24 h, fluorescence measurements were recorded according to Assay Instructions.

**Supplementary Figure-2:** VIP alone does not increase the CSC frequency: Indicated parental cells were either treated with 100 nM of VIP or left untreated. After 24 h, single cell suspensions were stained with CD133 antibodies, or double stained with CD44 and CD24 antibodies and analyzed by flow cytometry. CSCs are shown in blue and the percentage of CSCs is indicated. Note that VIP did not increase the CSC frequency either by converting PC to CSC or by enhancing the proliferation of CSCs.

**Supplementary Figure-3:** Expression of VIP in CSC: Intracellular expression of VIP was measured and compared between parental cells and CSCs by quantitative real time PCR. Modest expression of VIP compared to parental cells (PC) was observed.

**Supplementary Figure-4:** Phosphorylation of endogenous BAD and overexpressed BAD behave similarly in response to treatment with inhibitors and neuropeptide: Spheres were infected with lentivirus expressing wt-BAD. Two days after infection, cells were placed in supplement-free basal DMEM medium for 3 h and treated with indicated inhibitors for 3 h. Fifteen minutes later, 100 nM VIP was added. Phosphorylation at S112 of endogenous BAD and overexpressed BAD was detected from whole cell lysates. The S112 blot was stripped and reprobed with total BAD antibodies. Note that both endogenous BAD and overexpressed BAD undergo BAD dephosphorylation by treatment with inhibitors and rephosphorylated by VIP.

**Supplementary Table-1:** p values for various comparisons of caspase activity and sphere formation: Cytoprotective effects of various neuropeptides on CSCs.

**Supplementary Table-2:** p values for various comparisons of caspase activity and sphere formation: VIP-induced cytoprotective mechanisms in CSCs.

**Supplementary Table-3:** p values for various comparisons of caspase activity and sphere formation: VIP-induced cytoprotection is abrogated by dominant negative PKI-GFP and N17Ras.

**Supplementary Table-4:** p values for various comparisons of caspase activity and sphere formation: Absence of BAD desensitizes CSCs from drug-induced apoptosis.
